# Vincent’s Angina

**Published:** 1916-04

**Authors:** Ray Karchmer Daily

**Affiliations:** Houston, Texas


### TEXAS MEDICAL JOURNAL

**Dr. F. E. Daniel, Founder. Established July, 1885**

MRS. F. E. DANIEL, - Publisher and Managing Edito

Published Monthly.—Subscription, $1.00 a Year.

VOL. XXXI AUSTIN, APRIL, 1916 No. 10

**The publisher is not responsible for views of contributors**

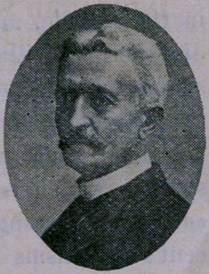

### Original Articles.

### Vincent's Angina.

BY DR. RAY KARCHMER DAILY, HOUSTON, TEXAS.

Vincent's angina is the local manifestation in the throat and mouth of a disease caused by the bacillus and spirillum of Vincent, and as in other parts of the body they may produce there a membranous; ulcerous or gangrenous process. It frequently follows the diseases of childhood,—measles, scarlet fever, diphtheria, whooping cough and other diseases, and as a secondary infection it is often very serious, destroying through sloughing large areas of tissue and causing a severe constitutional disturbance. As a primary infection it usually runs a mild course, and is by far more common than is generally supposed; probably many cases of it go undiagnosed, owing to the fact that the clinical symptoms are unreliable, and the diagnosis depends on bacteriologic examination.

The diagnosis of Vincent's angina must be made from a smear made directly from the lesion; a culture will not reveal the offending organism, as far as Vincent's organisms are concerned, because they do not grow in ordinary cultures; they have been cultured under anaerobic conditions, and even then the spirillae appear only after a long period of incubation. In a smear they are demonstrated readily with the ordinary stains, staining best with carbol-fuchsin; the spirillae decolorize rapidly with Gram's stain, the bacilli decolorize less easily and irregularly. A smear thus stained will show the fusiform bacilli and spirillae, and the combination leaves no doubt as to the diagnosis, and when once seen can hardly be mistaken for anything else ever afterwards.

The bacilli are thin, rod shaped with pointed ends, and swollen in the middle, and are about two or three times as long as the diphtheria bacillus; they do not stainly evenly and appear in stripes with deeper stain near the ends. The spirillae are slender spirals, coiled or twisted, varying in length and number of convolutions; they are easily differentiated from the spirochete of syphilis, which is smaller, extremely slender, has a characteristic cork-screw arrangement, and stains with great difficulty. Vincent's organisms may appear single, in groups or great clusters; in addition to the typical organisms, one may meet in a smear many other forms of the organisms not truly typical. A few cases of diphtheroid angina have been reported where the spirochete was absent, the bacillus being found alone. These cases are very rare, however, and the spirillum is always found in the deeper forms of Vincent's angina. Many laboratory investigators believe that the spirillum and bacillus are one organism, the spirillum being the adult form of the bacillus. Vincent opposes this theory, and with many others believes that they are two distinct organisms acting in symbiosis.

It is important to diagnose this condition from a medical as well as a surgical standpoint; it has to be differentiated from diphtheria, because the administration of antitoxin would only lower the general resistance without any beneficial effect. It also should not be mistaken for syphilis, because mercurialization increases the virulence of the infection and in several cases produced extensive sloughing. Surgically, it is important to know that there are none of these organisms in the oral cavity before operating; they have a tendency to invade the operated site and cause extensixe necrosis.

The conditions predisposing to Vincent's angina are general bad health and an unhealthy condition of the gums and teeth. The disease is communicable, but is dependent upon close contact. One of my.patients was a nursing mother, and ten days after her infection she brought to me the babe, who had a profuse sanguinary discharge from the nose; the infant had a small ulcer on the septum, and in the discharge were found Vincent's organisms. The eating and drinking utensils of the patient should be sterilized and their mouth discharges burned. The throat, mouth and teeth should receive special attention following an attack; the organisms are found around the gums and teeth for months after an attack, and reinfection of patients is exceedingly common.

The symptoms are interesting for their variability; the onset, as a rule, is sudden. A chill often ushers in the infection. The temperature varies from almost no increase at all to 104 or higher. The local symptoms vary from a small ulceration on one tonsil to a pseudomembrane extending over the tonsils, the uvula and palate, and passing on to necrosis. Adults complain of more local pain and greater malaise, than in follicular tonsilitis. The following cases will serve to illustrate the variability of the symptoms:

Case 1.—G. H., 8 years old, complained for several days of headache, pain in the throat and dysphagia; temperature, 100; on the right tonsil was a large deep puuched out ulcer, very suggestive of syphilis. The edges were hard and everted, the base rough and covered with a dirty brownish-gray exudate, which on rubbing had no tendency to bleed. A smear made from the ulcer showed the fusiform bacilli and spirochetes in great numbers. The ulcer had no tendency to spread and yielded in two weeks to local applications of 50 per cent silver nitrate and potassium chlorate gargle.

Case 2.—Mrs. C., 27 years old, for years has been subject to attacks of tonsilitis. Was recovering from a puerperal infection, and had no temperature for thee weeks. She suddenly had a chill, began to ache all over, and developed a sore throat; the pain in the throat kept her awake at night. Temperature 100.5, pulse 120, general appearance that of great depression. Both tonsils were large and red; right tonsil was covered with a shining dark-green membrane, not easily removed, and leaving a bleeding surface when pulled away. Diagnosis made from a smear, which consisted of large bunches of spirillae and bacilli of Vincent. The exudate spread and on the next day covered the tonsils, uvula and soft palate; the submaxillary glands were large; the odor fetid. Two days later she became hoarse, and for ten days could not speak above a whisper. The larynx had the typical appearance of an acute laryngitis; no ulceration or membrane could be seen. The exudate in the throat disappeared in a week, leaving large ulcers on the tonsil, and a small ulcer on the uvula, which healed in about three weeks.

Case 3.—E. R., 8 years old, complains of sore throat and difficulty of swallowing, temperature 100.4. The right tonsil had a foul-smelling cavity, with hard edges, with little exudate, bleeding freely upon touching with a cotton probe. No constitutional symptoms.

CASE 4.—E. S., 5 years old; referred for examination of throat, because she had been feverish for several days; child perfectly comportable. On the left tonsil were three round deposits of a soft pultaceous exudate, wiping off easily, and leaving an eroded area; the child had an ulcer on the left cheek and the gums were bleeding freely upon touch with a cotton-tipped probe. Diagnosis made from smear.

CASE 5.—Mrs. O., 32 years old. For several days had a sore throat, and aching pains all over the body. She had a chill and for several days was very hot. Temperature 101, pulse rapid; both tonsils had deep cavities, covered with a membrane, which on removing left a freely bleeding surface; the appearance was very suspicious of diphtheria. A smear showed Vincent's angina and a few streptococci. A culture showed streptococci.

CASE 6.—Mrs. A., 34 years old. Several days ago she had a chill, which was followed by pains over the body and a severe sore throat; was vomiting; had diarrhea; cervical glands enlarged; temperature 104, pulse 140; both tonsils were large and covered with a soft greenish-gray exudate involving the uvula, and bleeding very freely. I made the diagnosis of Vincent's angina and swabbed the affected area daily with 50 per cent silver nitrate, and gave the patient a potassium chlorate gargle. The exudate began to disappear, but for three days there was no improvement in her constitutional symptoms, which continued to be very severe. I then made a culture for diphtheria, which was reported negative. A smear made at that time threw no light on the case, and consisted of shreds of tissue with a few streptococci; the symptoms began to subside in a week. I still consider this a case of Vincent's angina, and believe that the silver nitrate interfered with the demonstration of the organism.

Every one of the adults affected complained of severe local pain, and had marked constitutional depression. The constitutional symptoms in children were slight.

The blood changes of Vincent's angina are interesting; a pronounced case shows a relative lymphocytosis in a differential count. Dr. F. E. Sanders reported that in two of his fatal cases the blood picture gradually became that of acute leukemia, the leucocyte count not being very high.

The prognosis in primary cases, as a rule, is good, unless it involves the larynx or trachea. Most cases yield in a few days; some become chronic and run for months. In secondary cases with a depleted general system the prognosis is not so favorable.

Treatment is principally local and symptomatic. Many remedies have been used locally; trichloracetic acid, iodine, chromic acid, silver nitrate, and others. My cases have yielded best to applications of 50 per cent silver nitrate and potassium chlorate gargles. Of late years salvarsan has been used, applied locally to the lesion, and intravenously. Many serious cases have been reported cured by this remedy, when all others failed.

